# Anatomy and Physiology of the Spinal Accessory Nerve

**Published:** 1843-12

**Authors:** 


*Anatomy and Physiology of the Spinal Accessory Nerve.*—A Sig. Morganti, of ihe University of Pavia, publishes the following, as the results of various experiments which he has performed on different animals with the view of discovering the certain origins, and accordingly the functions, of the spinal accessory nerve of Willis. His conclusions are:—

1. That the spinal accessory is a motor nerve.
2. By its external branch it conveys the power of motion to the muscle to which it is distributed.
3. By its internal branch it gives motion to the muscles of the larynx, and consequently *it is the nerve which serves for the production of voice.*
4. Its external branch is composed of fibrils, which proceed from the lowest origins of the nerve in the spinal cord.
5. Its internal branch is formed of the higher fibrillæ—those which originate next beneath the pneumogastric nerve—and contributes those fibrils to the latter nerve which form the recurrent nerve and *the motor fibres of the pneumogastric throughout its course.*—*Ibid*, from *Annali Universali di Medicina*, July, and Sept., 1843.

